# Simultaneous utilization of glucose and xylose for lipid production by *Trichosporon cutaneum*

**DOI:** 10.1186/1754-6834-4-25

**Published:** 2011-08-24

**Authors:** Cuimin Hu, Siguo Wu, Qian Wang, Guojie Jin, Hongwei Shen, Zongbao K Zhao

**Affiliations:** 1 Dalian Institute of Chemical Physics, CAS, Dalian 116023, China; 2 Dalian National Laboratory for Clean Energy, Dalian 116023, China; 3 Graduate University of the Chinese Academy of Sciences, Beijing 100049, China

## Abstract

**Background:**

Biochemical conversion of lignocellulose hydrolysates remains challenging, largely because most microbial processes have markedly reduced efficiency in the presence of both hexoses and pentoses. Thus, identification of microorganisms capable of efficient and simultaneous utilization of both glucose and xylose is pivotal to improving this process.

**Results:**

In this study, we found that the oleaginous yeast strain *Trichosporon cutaneum *AS 2.571 assimilated glucose and xylose simultaneously, and accumulated intracellular lipid up to 59 wt% with a lipid coefficient up to 0.17 g/g sugar, upon cultivation on a 2:1 glucose/xylose mixture in a 3-liter stirred-tank bioreactor. In addition, no classic pattern of diauxic growth behavior was seen; the microbial cell mass increased during the whole culture process without any lag periods. In shake-flask cultures with different initial glucose:xylose ratios, glucose and xylose were consumed simultaneously at rates roughly proportional to their individual concentrations in the medium, leading to complete utilization of both sugars at the same time. Simultaneous utilization of glucose and xylose was also seen during fermentation of corn-stover hydrolysate with a lipid content and coefficient of 39.2% and 0.15 g/g sugar, respectively. The lipid produced had a fatty-acid compositional profile similar to those of conventional vegetable oil, indicating that it could have potential as a raw material for biodiesel production.

**Conclusion:**

Efficient lipid production with simultaneous consumption of glucose and xylose was achieved in this study. This process provides an exciting opportunity to transform lignocellulosic materials into biofuel molecules, and should also encourage further study to elucidate this unique sugar-assimilation mechanism.

## Background

Lignocellulosic biomass such as forest and agricultural residue is the most abundant and renewable organic material in the biosphere. Using lignocellulose as a feedstock to produce biofuels and commodity chemicals is of great economic and environmental significance [1]. However, many problems must be solved before such ideal feedstock can be processed efficiently through biological routes. The sugar streams produced upon hydrolysis of lignocellulose are mixtures of hexoses and pentoses, mostly glucose and xylose, with a typical mass ratio around 2:1 [2]. Unless both the glucose and xylose are utilized, the economics of converting lignocellulosic biomass into bio-based products are unfavorable [3-5].

Microorganisms generally metabolize sugars sequentially when exposed to a mixture of glucose and xylose, because glucose can repress the utilization of other sugars via a catabolite repression mechanism or allosteric competition for sugar transporters [6]. A classic pattern of diauxic growth occurs when cells are exposed to multiple carbon sources in the presence of glucose, with a lag period occurring between growth phases [7]. Such physiological phenomena present a major disadvantage for biotransformation of biomass hydrolysates, which routinely contain various monosaccharides.

Recently, microbial lipid has been suggested as an alternative feedstock for biodiesel production, because making lipids by oleaginous microorganisms is potentially independent of arable land, and is continuous and controllable [8-10]. Utilization of glucose and xylose simultaneously is an important process in the utilization of lignocellulosic biomass and related raw materials as the carbon sources, in order to reduce the costs of microbial lipid technology. However, early studies have indicated that oleaginous yeasts convert sugar mixtures sequentially. *Rhodotorula toruloides *assimilated glucose first during cultivation in a mixture of glucose, xylose, xylulose and xylitol [11]. Similarly, in recent years, *Lipomyces starkeyi *[12], *Trichosporon fermentans *[13] and *R. glutinis *[14] have also been assessed for the production of lipid using carbon sources containing glucose and xylose. Although both hexose and pentose were converted into lipid in those examples, the sequential manner of substrate uptake significantly increased the cultivation time.

To continue our efforts on microbial lipid production using cheap feedstocks, we were interested in identifying strains capable of utilizing sugar mixtures more efficiently. In this scenario, we identified the *Trichosporon cutaneum *strain AS 2.571 as an oleaginous yeast that can assimilate glucose and xylose simultaneously. In this report, we detail our results on microbial lipid production by this strain using a mixture of glucose and xylose.

## Methods

### Yeast strain and cultivation conditions

The oleaginous yeast *T. cutaneum *AS 2.571 (China General Microbiological Culture Collection Center, Chinese Academy of Sciences, Beijing, China), stored in 20% glycerol at -80°C before use. The yeast strain was routinely cultivated in yeast extract/peptone/dextrose (YPD) broth (1% peptone, 1% yeast extract, 2% glucose) for inoculum preparation. The semi-defined medium contained carbon sources (glucose or D-xylose, or a mixture of both) 70 g/L, nitrogen sources (yeast extract 0.75 g/L and NH_4_Cl 0.1 g/L), MgCl_2_·6H_2_O 1 g/L,, Na_2_SO_4 _0.1 g/L, and phosphate buffer (KH_2_PO_4 _11.8 g/L, and K_2_HPO_4_·3H_2_O 3.7 g/L) unless otherwise stated. Trace element solution was added to all media separately after sterilization, to a final concentration of CaCl_2_·2H_2_O 40 mg/L, FeSO_4_·7H_2_O 5.5 mg/L, citric acid·H_2_O 5.2 mg/L, ZnSO_4_·7H_2_O 1.0 mg/L, MnSO_4_·H_2_O 0.76 mg/L and 18 mol/L H_2_SO_4 _1.84 × 10^-3 ^mg/L [15]. Pre-cultures were inoculated from fresh agar slants (one loopful) and grown at 30°C for 24-26 hours, then cultivation was initiated by transferring the pre-cultivated cell suspension into the lipid-production medium at 10% (v/v). All experiments were carried out at 30°C.

### Shake-flask cultivation in semi-defined media

Experiments were carried out in 250-mL unbaffled conical flasks with a working volume of 50 mL, at a rotation rate of 200 rpm at 30°C. Experiments were carried out with 70 g/L total sugars with different glucose:D-xylose mass ratios: 2:1, 1:1, 1:2, 1:0 (glucose only) and 0:1 (D-xylose only). All experiments were conducted for 120 hours. To test the possibility that supplementation of one sugar might change the outcome of the culture initiated with the other sugar as the sole carbon source, the experiment was started using a culture containing 35 g/L glucose or D-xylose as the sole carbon source, with an equal amount (35 g/L) of the other carbon source being added after 24-h cultivation.

### Batch cultivation in a 3-liter bioreactor

Batch cultivation was performed at 30°C in a 3-liter stirred-tank bioreactor (Biotech-2002, Shanghai Baoxing Bioengineering Equipment Co. Ltd., China) with a working volume of 2 liters. The cell suspension was inoculated into semi-defined medium with 47 g/L glucose and 23 g/L D-xylose. The phosphate-buffer solution was replaced by 0.4 g/L KH_2_PO_4_, and the pH was maintained at 6.0 by automatic addition of 2 mol/L NaOH. Cultures were stirred at 400 rpm and aerated with 0.4 vvm (volume of air per volume of liquid per minute; 0.8 L/min) air flow. Silicone antifoam (XPJ 900, Saiouxinyue Defoamer Co., Ltd., Jiangsu province, China) was supplemented when necessary.

### Preparation of corn-stover hydrolysate and fermentation in shake flasks

Corn stover (CS) collected from a local farm (Henan, China) was used as the raw material, and was dried and milled to a small size (1 to 4 mm). Dilute sulfuric-acid pretreatment and enzymatic hydrolysis were carried out following the method of Wang *et al*. [16]. The hydrolysate was concentrated, given an excess of lime with calcium hydroxide and filtered. The final hydrolysate contained 60 g/L total sugar, and was used for fermentation without any nutrient supplementation. The experiments were conducted at 30°C with pH 6.0.

### Analytical methods

The microbial cell mass, expressed as dry cell weight (DCW), was obtained from the cell pellet in 30 mL of culture broth. Cells were harvested by centrifugation (10,000 *g*) for 10 minutes at room temperature, washed with 0.9% w/v sodium chloride solution, and dried in an oven at 105°C to a constant weight.

In the semi-defined media, glucose concentration was quantified with a glucose analyzer (SBA-50B; Shandong Academy of Sciences, Jinan, China), and D-xylose concentration was obtained by subtracting glucose from the total reducing sugars, which were determined spectrophotometrically using the dinitrosalicylic acid (DNS) method [17]. Because of its complexity, the CS hydrolysate was analyzed for the concentrations of glucose and D-xylose by ion chromatography (IC) at 30°C (Dionex, Sunnyvale, CA USA) with a CarboPac PA10 analytical column, a CarboPac PA10 guard column and an ED50 electrochemical detector. The mobile phase (1 mL/min) was 22 mmol/L NaOH. Under these conditions, glucose and xylose typically had a retention time of 9.5 minutes and 10.5 minutes, respectively. Sugar-consumption rates were obtained by dividing the consumed sugar (g/L) by the time taken for the process to complete.

Total lipid was extracted using chloroform and methanol as previously described [18]. Lipid content was expressed as gram lipid per gram dry cell mass, and lipid coefficient as gram lipid produced per gram substrate consumed. The fatty acid (FA) compositional profiles of lipid samples were determined using gas chromatography (GC) (7890F) (Techcomp Bio-Equipment Co. Ltd., Shanghai, China) after transmethylation, according to the previously published procedure [19].

All reported data were averaged from experiments performed at least in duplicate.

## Results

### Utilization of a glucose:xylose mixture for lipid production by *T. cutaneum*

The experiments started with cultivation of *T. cutaneum *using a mixture of 47 g/L glucose and 23 g/L xylose as the carbon source in a phosphate-buffered solution at pH 5.5 to 6.0. Small amounts of yeast extract and NH_4_Cl were included in the medium to achieve an initial carbon:nitrogen (C:N) ratio of 351, a value very close to the optimal value of 420 for lipid production by the yeast *R. toruloides *[20]. After cultivation of *T. cutaneum *for 120 hours, the culture produced 23.8 g/L cell mass with a cellular lipid content of 49.7%, comparable with earlier results in which both glucose and xylose were present in the medium [12,21]. Although a higher C:N ratio favors lipid accumulation [20,21], further increases in the C:N ratio led to a much slower cell growth and almost no further improvement in cellular lipid content (data not shown).

### Simultaneous utilization of glucose and xylose by *T. cutaneum*

**Figure 1:**
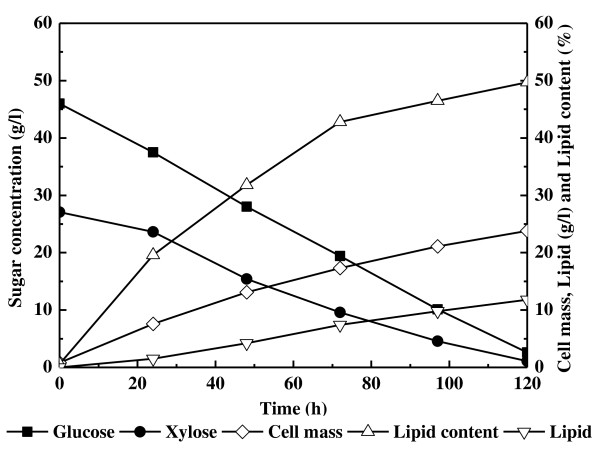
**Profiles of substrate consumption, cell-mass formation and lipid accumulation by *Trichosporon cutaneum***. Cells were cultivated in shake flasks on a substrate containing a mixture of glucose (47 g/L) and xylose (23 g/L).

To elucidate the substrate-assimilation profile by *T. cutaneum *using sugar mixtures as the carbon source, the time course of cell performance was determined (Figure 1). The initial culture was prepared based on a sugar mass containing 47 g/L glucose and 23 g/L xylose. However, because the xylose concentration in the mixture was estimated by subtracting glucose (determined by the glucose analyzer) from the total sugar (determined by the DNS method), it appeared to be higher than expected. The results indicated that the concentrations of glucose and xylose decreased over time, and they were 2.6 and 1.1 g/L, respectively, when the cultivation was stopped after 120 hours. Both sugars were assimilated simultaneously rather than sequentially. No diauxic behavior was seen; the cell mass and lipid increased without any lag period during the whole process.

**Figure 2:**
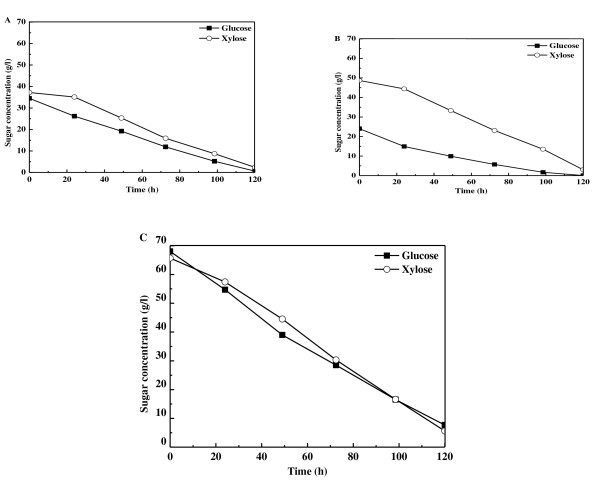
**Profiles of substrate consumption by *Trichosporon cutaneum *cultivated in shake flasks on glucose and xylose mixtures with a total sugar concentration of 70 g/L in different mass ratios**. **(A) **Glucose:xylose 1:1; **(B) **glucose:xylose 1:2; **(B) **glucose or xylose as the sole carbon source.

Additional culture experiments were conducted, in which glucose and xylose were mixed with different mass ratios, and the substrate-consuming curves plotted (Figure 2). Glucose and xylose were consumed in a simultaneous pattern when the glucose:xylose mass ratio was 1:1 (Figure 2A) or 1:2 (Figure 2B), in a similar manner to that shown in Figure 1. These data are in sharp contrast with the sequential utilization of glucose and xylose reported previously [22,23].

The results of lipid production by *T. cutaneum *using the glucose:xylose mixture are summarized in Table 1. The cellular lipid content, amount of lipid and lipid coefficient were 52.4%, 12.0 g/L and 0.20 g/g, respectively, when glucose was the sole carbon source. However, when xylose was the carbon source, these figures reduced to 46.5%, 9.9 g/L and 0.16 g/g, respectively (*p *< 0.05). When glucose:xylose mixtures were used (Table 1), both the cellular lipid content and the amount of lipid produced were slightly lower than those achieved with glucose, but higher than those with xylose. Increasing the proportion of xylose in the substrate decreased lipid production slightly. Evans *et al*. reported that xylose was a better substrate for lipid production in terms of lipid coefficient [24]. By contrast, lipid production by *T. cutaneum *using glucose clearly gave a slightly higher lipid coefficient than using either xylose alone or a glucose:xylose mixture as the carbon source (*p *< 0.05).

**Table 1:** Results of lipid production by *Trichosporon cutaneum *cultivated on glucose and xylose mixtures

Experiment number	Glucose:xylose, g/L	Culture time, hours	Cell mass, g/L	Lipid content, %	Lipid concentration, g/L	Lipid productivity, g/L/h	Lipid coefficient, g/g	Sugar-consumption rate, g/L/h		Relative proportions of fatty acids in lipid, %, w/w				
								
								Glucose	Xylose	C16:0	C16:1	C18:0	C18:1	C18:2
1	70:0	120	22.9	52.4	12.0	0.10	0.20	0.50	-	48.6	0.6	19.2	31.7	ND^a^
2	47:23	120	23.8	49.7	11.8	0.098	0.17	0.36	0.22	44.4	0.6	16.6	38.4	ND
3	35:35	120	23.0	50.3	11.6	0.097	0.17	0.28	0.29	44.4	0.6	17.6	36.9	0.5
4	23:47	120	23.2	48.4	11.2	0.094	0.16	0.20	0.38	43.4	0.6	16.5	39.0	0.5
5	0:70	120	21.2	46.5	9.9	0.082	0.16	-	0.50	44.1	0.7	16.7	38.1	0.5
6^b^	47:23	80	22.0	59.1	13.0	0.16	0.17	0.57	0.35	27.8	0.8	20.2	48.2	3.0
7^c^	36:25	96.5	19.3	39.2	7.6	0.078	0.15	0.36	0.17	34.0	2.4	6.7	53.5	3.4

^a^Not detected.

^b^Experiment in 3-liter bioreactor.

^c^Experiment using corn-stover hydrolysate.

Moreover, as shown in Table 1, sugar-consumption rates were roughly proportional to their individual concentrations in the medium, which led to complete consumption of both sugars at about the same time (Figure 2A-C). This phenomenon has also been reported recently during glucose and xylose co-fermentation by *Sulfolobus acidocaldarius *[3]. When glucose or xylose at 70 g/L was used as the sole carbon source, the substrate-consumption curves almost overlapped (Figure 2C), and exhibited the same sugar-consumption rate of 0.50 g/L/h (Table 1). When the two sugars were mixed to give a total sugar concentration of 70 g/L, both glucose and xylose were each assimilated at a reduced rate; for example, a 1:1 mixture resulted in a consumption of 0.28 g/L/h glucose and 0.29 g/L/h xylose. The total sugar-consumption rates were nearly identical regardless of which substrate compositions were (Table 1, experiments 1 to 5).

### The effect of sugar supplementation on substrates utilization

**Figure 3:**
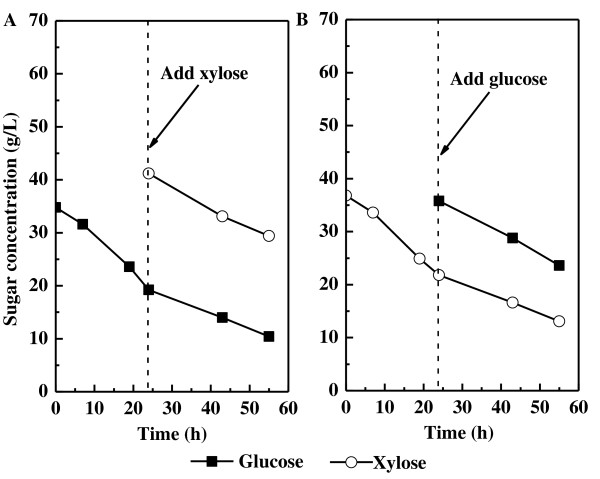
**Sugar-consumption profiles for the sugar-supplementation experiments**. **(A) **Xylose added into glucose-containing medium and **(B) **glucose was added into xylose-containing medium, after 24 hours of cultivation.

To further investigate the relationship between glucose and xylose consumption, the cultures initiated with xylose or glucose as the sole carbon source were supplemented after 24 hours with the other sugar (that is, glucose added to the xylose culture, and vice versa). For the initial 24-hour cultivation on 35 g/L glucose, the average glucose-consumption rate was 0.65 g/L/h (Table 2). Xylose was then introduced to give a final concentration of 35 g/L at 24 h. During the next 31 hours (the period from 24 to 55 hours), xylose was consumed at an average of 0.38 g/L/h, whereas the glucose consumption rate decreased to 0.28 g/L/h (Figure 3A; Table 2). However, the total sugar-consumption rate was 0.66 g/L/h, which was nearly equal to the rate before xylose supplementation. Similar results were obtained when glucose was introduced after 24 hours to the culture initiated with xylose (Figure 3B; Table 2,); the glucose was consumed at 0.39 g/L/h and the xylose-consumption rate reduced from 0.63 g/L/h to 0.28 g/L/h. The total sugar-consumption rate of 0.67 g/L/h was very close to the rate before glucose supplementation. These results indicated that pre-cultivation of *T. cutaneum *on one sugar had no discernible effects on its capacity to utilize the other sugar (*p *< 0.05), and confirmed that *T. cutaneum *assimilated glucose and xylose at rates proportional to their individual concentrations in the medium.

**Table 2:** The average sugar-consumption rates (g/L/h) of the sugar-supplementation experiments

Culture strategy	Before supplementation (0 to 24 hours)	After supplementation (24 to 55 hours)		
		
		Glucose	Xylose	Total
Initiated with 35 g/L glucose, then 35 g/L xylose added after 24 hours	0.65	0.28	0.38	0.66
Initiated with 35 g/L xylose, then 35 g/L glucose added after 24 hours	0.63	0.39	0.28	0.67

### Batch cultivation in a 3-liter bioreactor

**Figure 4:**
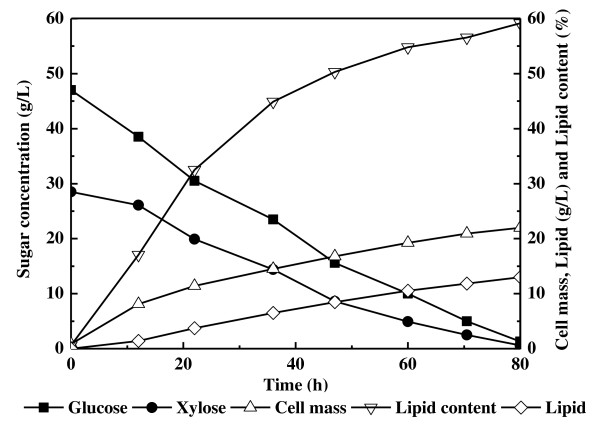
**Kinetics of lipid production by *Trichosporon cutaneum *cultivated in a 3-liter bioreactor on a mixture of glucose (47 g/L) and xylose (23 g/L)**.

To confirm our observation on simultaneous utilization of glucose and xylose obtained with shake-flask cultures, we cultivated *T. cutaneum *in a 3-liter stirred-tank bioreactor on a mixture of 47 g/L glucose and 23 g/L xylose. Samples were taken at different time intervals and analyzed. The results showed that the glucose and xylose were depleted simultaneously, and the carbon source was exhausted after 80 hours (Figure 4). The process was much faster than in the cultures using shake flasks, as indicated by the total sugar-consumption rate of 0.92 g/L/h, which might be the result of better control of the pH of the medium and better oxygen supply in the bioreactor unit. The cell mass and cellular lipid content increased gradually with final values of 22.0 g/L and 59.1%, respectively (Table 1). Lipid concentration was 13.0 g/L, which was also higher than that obtained in the shake-flask cultures (Table 1). More significantly, lipid productivity reached 0.16 g/L/h, which was over 1.6 times higher. These results were significantly better than previous studies with other oleaginous microorganisms using glucose and xylose as the carbon source [12-14].

### Lipid production with corn-stover hydrolysate

**Figure 5:**
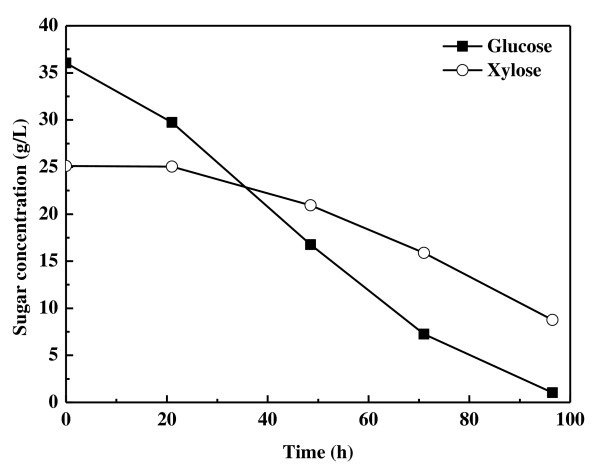
**Profiles of sugar consumption by *Trichosporon cutaneum *cultivated in corn-stover hydrolysate**.

CS hydrolysate was detoxified with an excess of lime, and used for lipid production directly. After 96.5 hours of cultivation, *T. cutaneum *had consumed 35.1 g/L glucose and 16.3 g/L xylose, and the residual sugars were 8.8 g/L xylose and 1.0 g/L glucose. The sugar-consumption profile indicated that glucose and xylose were consumed simultaneously, not sequentially, although xylose was used at a lower rate (Figure 5). The lipid concentration, lipid content and lipid coefficient were 7.6 g/L, 39.2% and 0.15 g/g, respectively (Table 1). The lipid productivity (0.078 g/L/h) and sugar-consumption rate (0.53 g/L/h) were similar to those obtained with xylose.

### Fatty-acid compositional data

The lipid produced by *T. cutaneum *was transmethylated and the corresponding FA methyl ester samples were analyzed by GC (Table 1). Palmitate (C16:0), stearate (C18:0) and oleate (C18:1) were the three predominant components in all samples, similar to lipid samples from most oleaginous yeasts [25]. This FA compositional profile is also similar to that of vegetable oil, suggesting that the lipid produced by this yeast could be a potential candidate for biodiesel production [10,26]. For those samples prepared using shake flasks in semi-defined media, no major changes were found in terms of FA composition, regardless of the glucose:xylose ratio. However, the samples from the 3-liter bioreactor culture and the culture using the CS hydrolysate had different compositional distributions of those three major FAs.

## Discussion

During the cultivation of *T. cutaneum *AS 2.571 on the glucose:xylose mixture at a mass ratio of 2:1, both sugars were assimilated simultaneously rather than sequentially. Cell mass and lipid accumulated without any lag period, in sharp contrast to the classic diauxic behavior. Substrate-consumption curves for different glucose:xylose mass ratios also exhibited a simultaneous pattern, and the substrate-consumption rate was roughly proportional to the individual concentrations of the sugars in the medium; the higher the initial sugar concentration, the higher the rate of sugar consumption. Glucose and xylose were consumed at an almost equal rate, both alone and when mixed together at a mass ratio of 1:1. Both glucose and xylose were almost completely utilized at the same time in all experiments starting with an initial total sugar concentration of 70 g/L in shake flasks, indicating that *T. cutaneum *took up glucose and xylose molecules simultaneously. Similar sugar uptake behavior for glucose and xylose has been reported for *S. acidocaldarius *[3]. The sugar-supplementation results indicated that, when the medium was supplemented with a second sugar, the sugar-consumption capacity was redistributed between the two sugars, which further confirmed the non-preference of the yeast when assimilating mixed sugars.

Xylose is generally transported by several different systems in yeasts. In *Candida shehatae*, a naturally xylose-utilizing yeast, there are at least two kinetically distinct xylose-transport systems [27,28]. The low-affinity transporter is generally shared with glucose, transporting sugar by a facilitated diffusion process driven by the concentration gradient. The high-affinity transporter is specific for xylose, which transports xylose together with a proton, using the proton motive force. Because *T. cutaneum *showed no preference between glucose and xylose, these two sugars may have their specific transporters functioning at similar efficiency in the lipid-production culture. Alternatively, a unique sugar transporter that has nearly identical affinity to both sugars may be operating in this yeast, as the individual sugar-consumption rate was closely related to the concentration of that sugar in the medium. However, the mechanism of simultaneous assimilation of glucose and xylose by *T. cutaneum *awaits further study.

Simultaneous utilization of glucose and xylose was also identified using real lignocellulosic hydrolysate, namely CS hydrolysate without any nutrition supplementation. The lipid coefficient and sugar-consumption rate were comparable with those obtained in the semi-defined media. However, the xylose-consumption rate was lower than expected based on its concentration, which led to incomplete consumption of xylose at the late stage of the cultivation. It is known that furfural and hydroxymethylfurfural present in lignocellulosic hydrolysate can be converted to their alcohol or acid derivatives by microorganisms through NAD(P)H/NAD(P)^+^-dependent redox reactions [29,30]. These processes may break the cofactor balance in xylose metabolism in yeasts in which NAD(P)H and NAD(P)^+ ^function during xylitol and xylulose formation [31]. In addition, other components in the hydrolysate may also influence the sugar-transport system. More detailed experiments are required to improve the culture performance for efficient utilization of lignocellulosic hydrolysate.

Microorganisms generally prefer glucose over other naturally occurring sugars. However, biomass, as it exists on earth, usually consists of glucose and other sugars. CS, for example, releases glucose, xylose and other monosaccharides after hydrolysis. As the predominant components, glucose and xylose can be present in biomass hydrolysates at mass ratios of 2:1 [2], and sometimes 1:2 or 1:1 [32], depending on the pretreatment and hydrolysis technology. Thus, strains that can utilize both glucose and xylose simultaneously and efficiently are crucial in terms of exploitation of biomass as feedstock. More importantly, elucidation of this unique sugar-assimilation mechanism should be of great importance in developing superior microorganisms for industrial biotechnology.

## Conclusion

In the present study, we found that the oleaginous yeast *T. cutaneum *AS 2.571 could assimilate glucose and xylose simultaneously to accumulate intracellularly a considerable amount of lipid with a good lipid coefficient, in both artificial and real hydrolysates. Our results provide an exciting process for biochemical conversion of lignocellulosic materials, as major hexoses and pentoses presented in the biomass hydrolysates can now be converted efficiently into lipid. More importantly, the absence of diauxic growth should encourage further study to elucidate this unique sugar-assimilation mechanism, which may help to develop superior microorganisms for industrial biotechnology.

## Competing interests

The authors declare that they have no competing interests.

## Authors' contributions

CH designed the study, performed the experiments, analyzed the results and wrote the manuscript. SW participated in the design of the study and commented on the manuscript. QW participated in the sugar analysis. GJ and HS participated in the bioreactor experiments. ZKZ coordinated the study and revised the manuscript. All authors participated in the correction of the manuscript, and approved the final version.

## References

[B1] Wisselink HW Toirkens MJ Wu Q Pronk JT van Maris AJA Novel evolutionary engineering approach for accelerated utilization of glucose, xylose, and arabinose mixtures by engineered *Saccharomyces cerevisiae *strains Applied And Environmental Microbiology 2009 75 4 907 914 10.1128/AEM.02268-08 19074603 PMC2643596

[B2] Ye XP Liu L Hayes D Womac A Hong KL Sokhansanj S Fast classification and compositional analysis of cornstover fractions using Fourier transform near-infrared techniques Bioresource Technology 2008 99 15 7323 7332 10.1016/j.biortech.2007.12.063 18249535

[B3] Joshua CJ Dahl R Benke PI Keasling JD Absence of diauxie during simultaneous utilization of glucose and xylose by *Sulfolobus acidocaldarius* Journal Of Bacteriology 2011 193 6 1293 1301 10.1128/JB.01219-10 21239580 PMC3067627

[B4] Kim JH Block DE Mills DA Simultaneous consumption of pentose and hexose sugars: an optimal microbial phenotype for efficient fermentation of lignocellulosic biomass Applied microbiology and biotechnology 2010 88 5 1077 1085 10.1007/s00253-010-2839-1 20838789 PMC2956055

[B5] Nichols NN Dien BS Bothast RJ Use of catabolite repression mutants for fermentation of sugar mixtures to ethanol Applied microbiology and biotechnology 2001 56 1-2 120 125 10.1007/s002530100628 11499918

[B6] Kawaguchi H Vertes AA Okino S Inui M Yukawa H Engineering of a xylose metabolic pathway in *Corynebacterium glutamicum* Applied And Environmental Microbiology 2006 72 5 3418 3428 10.1128/AEM.72.5.3418-3428.2006 16672486 PMC1472363

[B7] Aduse-Opoku J Mitchell W Diauxic growth of *Clostridium thermosaccharolyticum *on glucose and xylose FEMS microbiology letters 1988 50 1 45 49 10.1111/j.1574-6968.1988.tb02909.x

[B8] Liu B Zhao ZK Biodiesel production by direct methanolysis of oleaginous microbial biomass Journal Of Chemical Technology And Biotechnology 2007 82 8 775 780 10.1002/jctb.1744

[B9] Meng X Yang JM Xu X Zhang L Nie QJ Xian M Biodiesel production from oleaginous microorganisms Renewable Energy 2009 34 1 1 5 10.1016/j.renene.2008.04.014

[B10] Zhao ZB Hua YY Liu B Strategies to secure feedstock supply for chinese biodiesel industry China Biotechnology 2005 25 11 1 6

[B11] Hsiao HY Chiang LC Ueng PP Tsao GT Sequential utilization of mixed monosaccharides by yeasts Applied And Environmental Microbiology 1982 43 4 840 845 6211144 10.1128/aem.43.4.840-845.1982 PMC241929

[B12] Zhao X Kong XL Hua YY Feng B Zhao ZBK Medium optimization for lipid production through co-fermentation of glucose and xylose by the oleaginous yeast *Lipomyces starkeyi* European Journal Of Lipid Science And Technology 2008 110 5 405 412 10.1002/ejlt.200700224

[B13] Huang C Zong MH Wu H Liu QP Microbial oil production from rice straw hydrolysate by *Trichosporon fermentans* Bioresource Technology 2009 100 19 4535 4538 10.1016/j.biortech.2009.04.022 19433350

[B14] Dai CC Tao J Xie F Dai YJ Zhao M Biodiesel generation from oleaginous yeast *Rhodotorula glutinis *with xylose assimilating capacity African Journal of Biotechnology 2007 6 18 2130 2134

[B15] Meesters PAEP Huijberts GNM Eggink G High-cell-density cultivation of the lipid accumulating yeast *Cryptococcus curvatus *using glycerol as a carbon source Applied microbiology and biotechnology 1996 45 5 575 579 10.1007/s002530050731

[B16] Wang Y Fermentation system research of biodiesel oil production from fibre saccharified liquid Henan: Henan Agricultural University 2008

[B17] Miller G Use of dinitrosalicylic acid reagent for determination of reducing sugar Analytical Chemistry 1959 31 3 426 428 10.1021/ac60147a030

[B18] Li ZF Zhang L Shen XJ Lai BS Sun SQ A comparative study on four method of fungi lipid extraction Microbiology 2001 28 6 72 75

[B19] Li YH Zhao ZK Bai FW High-density cultivation of oleaginous yeast *Rhodosporidium toruloides *Y4 in fed-batch culture Enzyme And Microbial Technology 2007 41 3 312 317 10.1016/j.enzmictec.2007.02.008

[B20] Li YH Liu B Zhao ZK Bai FW Optimization of culture conditions for lipid production by *Rhodosporidium toruloides* Chinese Journal Of Biotechnology 2006 22 4 650 656 10.1016/S1872-2075(06)60050-2 16894904

[B21] Zhu LY Zong MH Wu H Efficient lipid production with *Trichosporon fermentans *and its use for biodiesel preparation Bioresource Technology 2008 99 16 7881 7885 10.1016/j.biortech.2008.02.033 18394882

[B22] Cheng KK Cai BY Zhang JA Ling HZ Zhou YZ Ge JP Xu JM Sugarcane bagasse hemicellulose hydrolysate for ethanol production by acid recovery process Biochemical Engineering Journal 2008 38 1 105 109 10.1016/j.bej.2007.07.012

[B23] Kong XL Liu B Zhao ZB Feng B Microbial production of lipids by cofermentation of Glucose and xylose with *Lipomyces starkeyi *2# Chinese Journal of Bioprocess Engineering 2007 5 2 36 41

[B24] Evans CT Ratledge C A comparison of the oleaginous yeast, *Candida curvata*, grown on different carbon sources in continuous and batch culture Lipids 1983 18 9 623 629 10.1007/BF02534673 6633167

[B25] Johnson VW Singh M Saini VS Adhikari DK Sista V Yadav NK Utilization of molasses for the production of fat by an oleaginous yeast, *Rhodotorula glutinis *IIP-30 Journal of Industrial Microbiology 1995 14 1 1 4 10.1007/BF01570057

[B26] Tao J Dai CC Dai Q Zhao M The conversion efficiency and economic feasibility of microbial energy Journal Of Microbiology 2006 26 6 48 54

[B27] Does AL Bisson LF Characterization of xylose uptake in the yeasts *Pichia heedii *and *Pichia stipitis* Applied And Environmental Microbiology 1989 55 1 159 164 16347817 10.1128/aem.55.1.159-164.1989 PMC184071

[B28] Lucas C van Uden N Transport of hemicellulose monomers in the xylose-fermenting yeast *Candida shehatae* Applied microbiology and biotechnology 1986 23 6 491 495 10.1007/BF02346066

[B29] Almeida JRM Bertilsson M Gorwa-Grauslund MF Gorsich S Liden G Metabolic effects of furaldehydes and impacts on biotechnological processes Applied microbiology and biotechnology 2009 82 4 625 638 10.1007/s00253-009-1875-1 19184597

[B30] Liu ZL Molecular mechanisms of yeast tolerance and *in situ *detoxification of lignocellulose hydrolysates Applied microbiology and biotechnology 2011 90 3 809 825 10.1007/s00253-011-3167-9 21380517

[B31] Kotter P Ciriacy M Xylose fermentation by Saccharomyces cerevisiae Applied microbiology and biotechnology 1993 38 6 776 783 10.1007/BF00167144

[B32] Ko CH Chiang PN Chiu PC Liu CC Yang CL Shiau IL Integrated xylitol production by fermentation of hardwood wastes Journal Of Chemical Technology And Biotechnology 2008 83 4 534 540 10.1002/jctb.1828

